# Survival of women with pregnancy-associated breast cancer according to clinical characteristics: A propensity score matching study

**DOI:** 10.1097/MD.0000000000030831

**Published:** 2022-10-07

**Authors:** Hongki Gwak, Sang Seok Woo, Eun-Sook Lee, Min Ho Park, Seokwon Lee, Hyun Jo Youn, Seho Park, In Suck Suh, Seong Hwan Kim

**Affiliations:** a Division of Breast and Thyroid Surgical Oncology, Department of Surgery, St. Vincent’s Hospital, College of Medicine, The Catholic University of Korea, Suwon, Korea; b Department of Plastic and Reconstructive Surgery, Kangnam Sacred Heart Hospital, Hallym University College of Medicine, Seoul, Korea; c Center for Breast Cancer, Hospital and Research Institute, National Cancer Center, Goyang, Korea; d Chonnam National University Medical School and Chonnam National University Hwasun Hospital, Gwangju, Korea; e Department of Surgery, Biomedical Research Institute, Pusan National University Hospital, Busan, Republic of Korea; f Department of Surgery, Jeonbuk National University Medical School, Jeonju, Korea; g Division of Breast Surgery, Department of Surgery, Yonsei University College of Medicine, Seoul, Korea.

## Abstract

In recent years, postponing childbearing has increased the prevalence of pregnancy-associated breast cancer (PABC). PABC has a poorer prognosis than breast cancer not associated with pregnancy (non-PABC) due to delayed diagnosis and aggressive subtype. Additionally, pregnancy itself predicts a poor prognosis; but, this is a subject of debate. Thus, we analyzed the effects of known prognostic factors and pregnancy on the prognosis of PABC. We retrospectively analyzed women aged 20 to 49 years who were diagnosed with breast cancer (BC) between 1989 and 2014. Patients were distributed into PABC and non-PABC groups, and 1:4 propensity score matching was performed to adjust for baseline characteristics. Primary endpoints were overall survival (OS) and BC-specific survival (BCSS). Secondary endpoint was the difference in prognosis according to BC subtype. Of the 34,970 recruited patients with BC, 410 (1.2%) had PABC. Patients with PABC were younger and tended to have triple-negative BC (TNBC) subtype than non-PABC patients. The 1640 matched non-PABC patients showed a significantly worse mean survival rate than the unmatched non-PABC patients. Patients with PABC had a significantly worse OS and BCSS than those with non-PABC. In multivariate analyses, patients with PABC of luminal B (Ki-67 ≥14.0%) and TNBC subtypes had worse OS and BCSS than patients with non-PABC. Patients with PABC had poorer prognosis than non-PABC patients after adjusting for several prognostic factors. This difference was particularly significant in patients with the luminal B and TNBC subtypes.

## 1. Introduction

Pregnancy-associated breast cancer (PABC) is defined as breast cancer (BC) that occurs during pregnancy or within 1 year after delivery.^[1]^ As the age at childbirth increases, the incidence of PABC also increases.^[2–4]^ PABC is a complex disease affecting patients, their families, and clinicians. Treatment is challenging for clinicians because the goal is to ensure the safety of both the mother and baby.

Patients with PABC tend to be younger than breast cancer not associated with pregnancy (non-PABC) patients and often present with more advanced disease, leading to worse prognosis. However, some studies have suggested that pregnancy itself predicts a poor prognosis of PABC. These conclusions are controversial, but it is difficult to conduct randomized controlled studies due to the rarity of this disease and ethical issues.

We conducted a retrospective study using propensity score matching (PSM) to analyze the prognosis of PABC and evaluate effects of known prognostic factors.

## 2. Methods

### 2.1. Compliance with ethical standards

The study protocol was approved by the Institutional Review Board of the Catholic University of Korea (VC21ZADI0234; Seoul, South Korea). All patient data were collected by the Korean Breast Cancer Society. All patients provided written informed consent for the storage and use of their information for research purposes.

### 2.2. Data source and study population

This retrospective study was based on prospectively collected data from patients who underwent BC surgery at 102 general hospitals. Women aged 20 to 49 years who were diagnosed with BC and underwent surgery between 1989 and 2014 were included in the study. Patients without estrogen receptor (ER), progesterone receptor (PR), human epidermal growth factor receptor 2 (HER2), and Ki-67 results, that are necessary immunohistochemistry data for molecular subtype classification, and patients without stage results, were excluded. PABC was defined as a diagnosis of BC during pregnancy or within 1 year after delivery.

### 2.3. Subtypes

Immunohistochemistry was performed according to the local hospital practice. HER2-positivity was defined as either an immunohistochemical staining score of 3+ or HER2 amplification according to fluorescence in situ hybridization (HER2-to-chromosome centromere 17 ratio ≥2.0). Clinical and pathological tumor–node–metastasis stages were classified according to the sixth edition of the American Joint Committee on Cancer. Tumors were classified into the following five subtypes based on ER, PR, HER2, and Ki-67 statuses: luminal A (ER- and/or PR-positive, HER2-negative, and Ki-67 <14.0%), luminal B (hormone receptor-positive, HER2-negative, and Ki-67 ≥14.0%), luminal HER2 (hormone receptor-positive and HER2-positive), HER2 amplified (ER-negative, PR-negative, and HER2-positive), and triple-negative BC (TNBC; ER-negative, PR-negative, and HER2-negative).

### 2.4. Statistical analyses

Prior to statistical analyses, a logistic regression model was used to select factors potentially affecting patient outcomes, including age, surgical method, cancer stage, BC subtype, tumor pathological stage, and history of chemotherapy. The propensity score for each factor was calculated using a regression model, and the closest propensity score was based on a control ratio of 1:4 using the nearest-neighbor algorithm approach with no replacement. The chi-square test was used to compare baseline characteristics between the patient groups. Survival curves were generated using the Kaplan–Meier method, and survival differences were analyzed using the log-rank test. We calculated the hazard ratios and 95% confidence intervals for BC-specific survival (BCSS) and overall survival (OS) using the Cox proportional hazards model. Statistical analyses were performed using R software version 4.0.2 (R Core Team [2013] R: A language and environment for statistical computing. R Foundation for Statistical Computing, http://www.R-project.org/, Vienna, Austria). A *P* < .05 was considered to be statistically significant.

## 3. Results

### 3.1. Baseline characteristics

**Figure 1.**
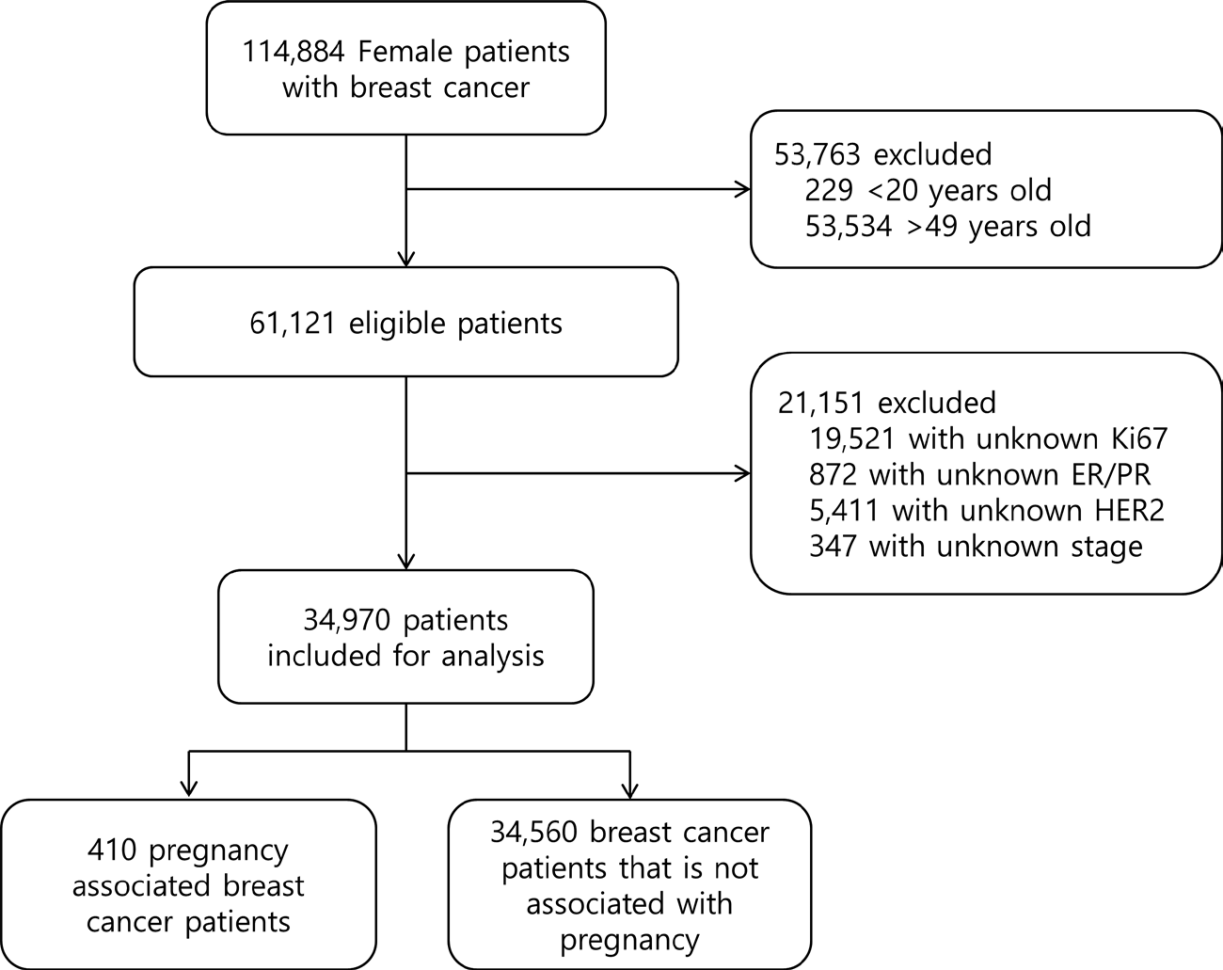
Flow chart of patient population. Data collection was performed between 1989 and 2014. ER = estrogen receptor, HER2 = human epidermal growth factor receptor 2, PR = progesterone receptor.

Of the 114,884 women with BC, 74,914 were excluded, and 34,970 were finally included in the analysis (Fig. 1). Of the patients included in the analysis, 410 (1.2%) had PABC. The PABC group was significantly younger than non-PABC group, with a mean age of 34.4 (±5.1) versus 42.2 (±5.3) years (*P* < .001). The number of patients aged <35 years was 60% in the PABC group, that was significantly higher than that in non-PABC group (9.8%) (*P* < .001). Patients with PABC had a significantly more advanced stage and higher histological grade than non-PABC patients. In non-PABC patients, stage I accounted for 39.6% and stage IV accounted for 1.4%; but, in PABC patients, stage I accounted for 22.4% and stage IV accounted for 5.6% (*P* < .001). The luminal A subtype had a significantly lower proportion (PABC 4.1% vs non-PABC 15.2%) and TNBC had significantly higher proportion in the PABC group (PABC 42.2% vs non-PABC 17.4%) than in non-PABC group (*P* < .001). Chemotherapy was administered significantly more frequently in the PABC group than in non-PABC group (89.5% vs 80.1%, *P* < .001). Variables showing a significant difference between the two groups, such as age, stage, tumor type, histologic grade, subtype, and chemotherapy, were corrected using PSM. The baseline characteristics of the patients before and after PSM are shown in Table 1.

**Table 1:** Clinical characteristics of patients before and after propensity-score matching.

Characteristics	Before propensity score matching, n (%)			After propensity score matching, n (%)		
	PABC (n = 410)	non-PABC (n = 34,560)	*P* value	PABC (n = 410)	non-PABC (n = 1640)	*P* value
Age						
<35	246 (60.0)	3396 (9.8)	<.001	246 (60.0)	982 (59.9)	1
≥35	164 (40.0)	31164 (90.2)		164 (40.0)	658 (40.1)	
Operation						
Breast-conserving	211 (51.5)	18,969 (54.9)	.178	211 (51.5)	888 (54.1)	.347
Mastectomy	199 (48.5)	15,591 (45.1)		199 (48.5)	752 (45.9)	
Stage*						
I	92 (22.4)	13,692 (39.6)	<.001	92 (22.4)	376(22.9)	.292
II	93 (22.7)	5019 (14.5)		93 (22.7)	375 (22.9)	
III	202 (49.3)	15,372 (44.5)		202 (49.3)	831 (50.7)	
IV	23 (5.6)	477 (1.4)		23 (5.6)	58 (3.5)	
Tumor type						
Ductal invasive	387 (94.4)	33,181 (96.0)	<.001	387 (94.4)	1551 (94.6)	.972
Lobular invasive	5 (1.2)	1055 (3.1)		5 (1.2)	21 (1.3)	
Other	18 (4.4)	324 (0.9)		18 (4.4)	68 (4.1)	
Histological grade†						
I	27 (7.8)	5184 (16.9)	<.001	32 (9.1)	131 (9.1)	.28
II	113 (32.6)	14,102 (46.1)		113 (32.1)	523 (36.5)	
III	207 (59.7)	11,321 (37.0)		207 (58.8)	779 (54.4)	
Unknown	63	3953		58	207	
Subtype‡						
Luminal A	17 (4.1)	5255 (15.2)	<.001	17 (4.1)	70 (4.3)	1
Luminal B	128 (31.2)	15,557 (45.0)		128 (31.2)	513 (31.3)	
Luminal HER2	43 (10.5)	4823 (14.0)		43 (10.5)	167 (10.2)	
HER2	49 (12.0)	2925 (8.5)		49 (12.0)	197 (12.0)	
TNBC	173 (42.2)	6000 (17.4)		173 (42.2)	693 (42.3)	
Hormone therapy						
Yes	150 (92.6)	22,556 (88.0)	<.001	150 (92.6)	627 (91.4)	.753
No	12 (7.4)	3079 (12.0)		12 (7.4)	150 (8.6)	
Chemotherapy						
Yes	367 (89.5)	27,668 (80.1)	<.001	367 (89.5)	1483 (90.4)	.577
No	43 (10.5)	6892 (19.9)		43 (10.5)	157 (9.6)	

HER2 = human epidermal growth factor receptor 2, non-PABC = breast cancer not associated with pregnancy, PABC = pregnancy-associated breast cancer, TNBC = triple-negative breast cancer.

* TNM classification according to the 6th edition of the American Joint Committee on Cancer.

† The grade according to Bloom–Richardson17 was taken from local pathology reports.

‡ Immunohistochemistry was performed according to local practice.

### 3.2. Survival analyses

**Figure 2.**
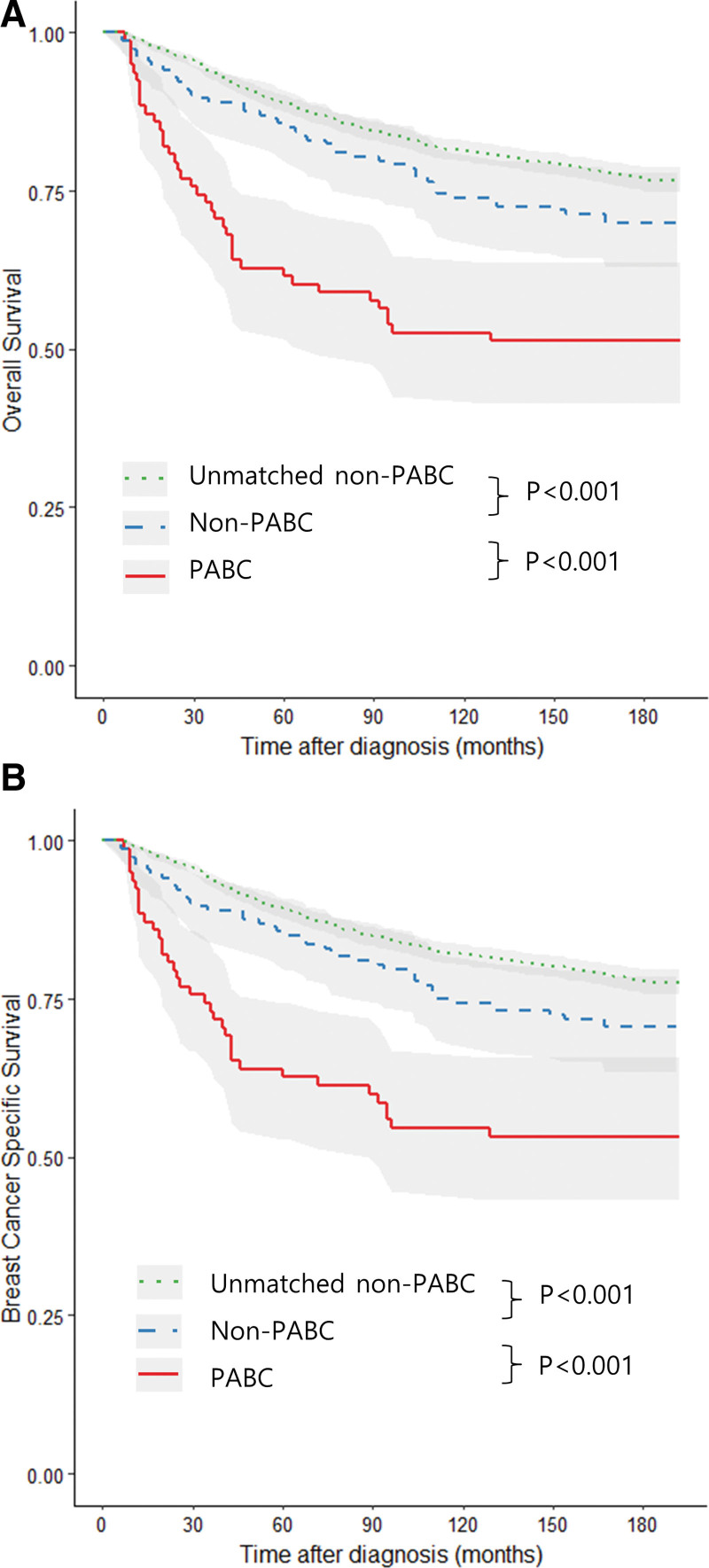
Kaplan–Meier curves for overall survival (A) and breast cancer specific survival (B).

The mean follow-up duration was 87 months. After PSM, the OS and BCSS of the non-PABC group were significantly poorer than those of unmatched non-PABC group (both *P* < .001). The OS and BCSS of the PABC group were significantly worse than those of matched non-PABC group (both *P* < .001) (Fig. 2).

### 3.3. Subgroup analyses

**Figure 3.**
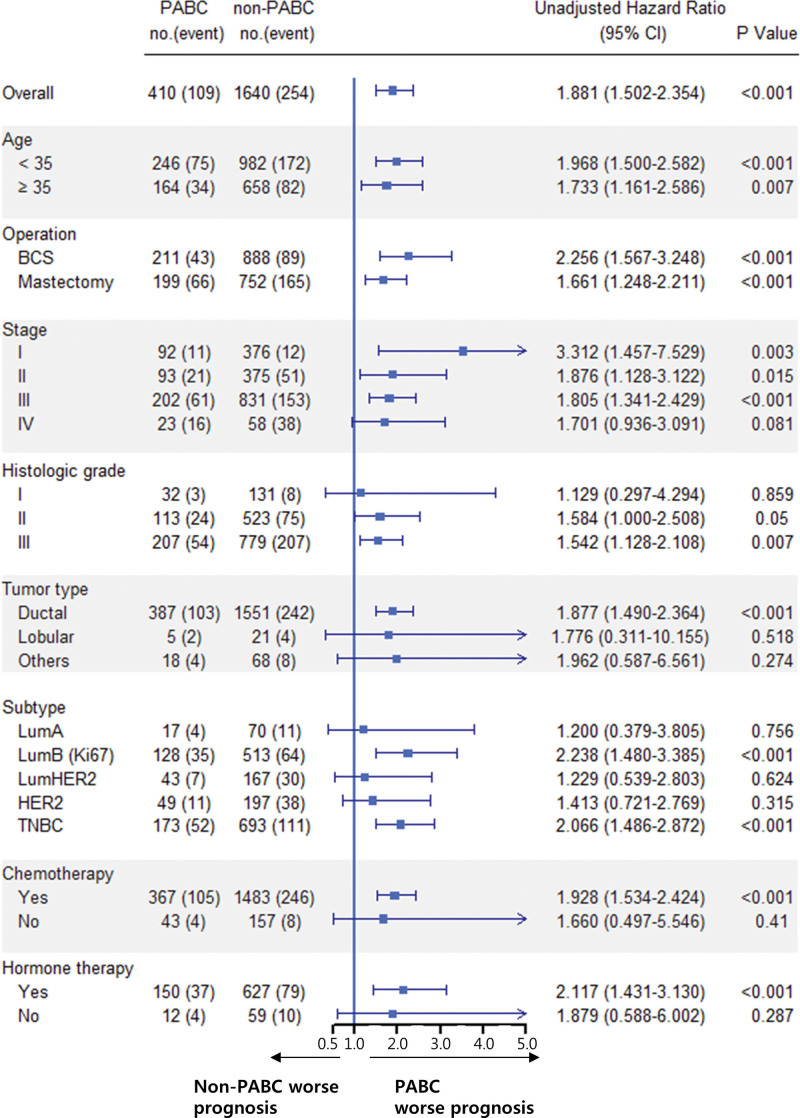
Forest plot of the association of pregnancy with death from any cause in various subgroups after propensity-score matching. PABC = pregnancy-associated breast cancer.

**Figure 4.**
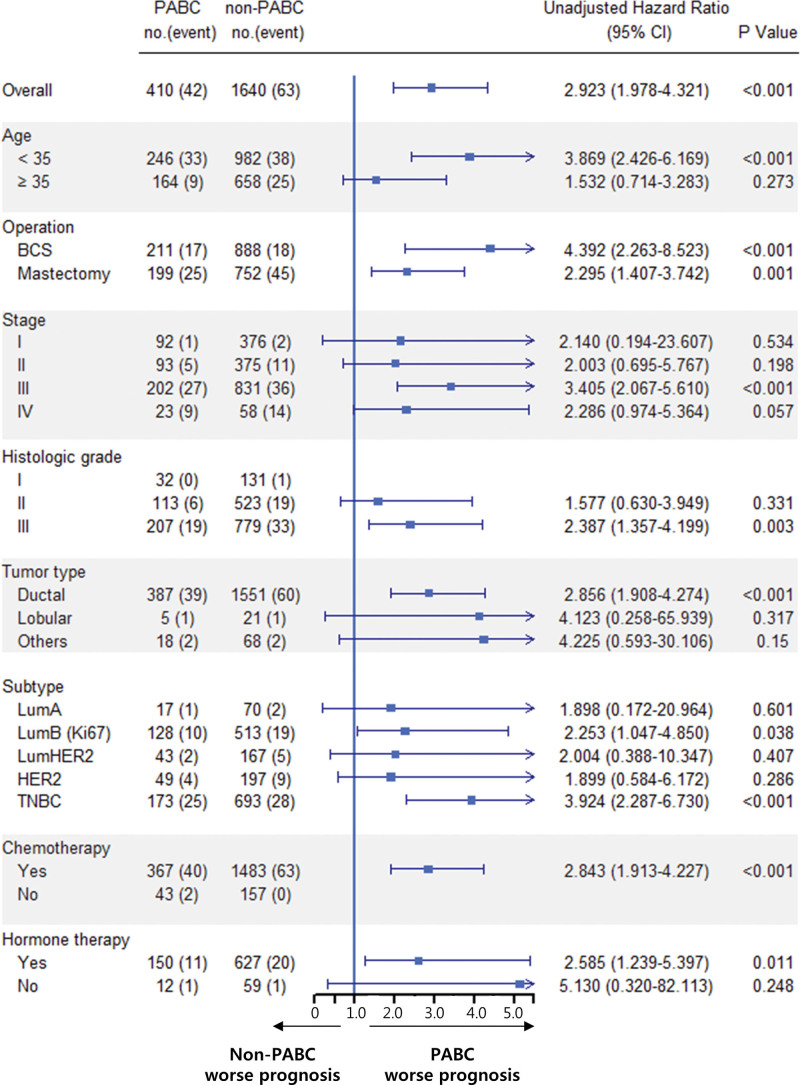
Forest plot of the association of pregnancy with death from breast cancer in various subgroups after propensity-score matching. PABC = pregnancy-associated breast cancer.

Cox regression analysis was performed on all variables used in the PSM. Patients with PABC showed unfavorable OS and BCSS for all variables in the univariate analysis (Figs. 3 and 4). Patients with PABC aged <35 years and with the luminal B or TNBC subtype had significantly worse OS and BCSS than the respective non-PABC patients (Figs. 3 and 4), consistent with the multivariate analyses (Table 2).

**Table 2:** Multivariate analyses of factors associated with the overall survival of breast-cancer-specific survival in pregnancy-associated breast cancer patients.

	Overall survival		Breast cancer-specific survival	
	Hazard ratio (95% CI)	*P* value	Hazard ratio (95% CI)	*P* value
Overall	1.652 (1.280–2.132)	**<.001**	1.620 (1.250–2.100)	**<.001**
Age				
<35	1.626 (1.146–2.307)	**.006**	1.594 (1.156–2.198)	**.004**
≥35	1.787 (1.076–2.967)	**.025**	1.773 (1.134–2.772)	**.012**
Operation				
BCS	1.812 (1.084–3.030)	**.023**	1.814 (1.161–2.833)	**.009**
Mastectomy	1.627 (1.144–2.314)	**.007**	1.576 (1.137–2.183)	**.006**
Stage				
I	3.553 (1.167–10.821)	**.026**	3.006 (1.131–7.986)	**.027**
II	2.358 (1.285–4.327)	**.006**	2.100 (1.226–3.598)	**.007**
III	1.342 (0.914–1.969)	.133	1.388 (0.983–1.960)	.063
IV	2.472 (0.830–7.364)	.104	1.682 (0.636–4.449)	.294
Tumor type				
IDC	1.668 (1.250–2.225)	**.001**	1.657 (1.275–2.153)	**<.001**
ILC	NA	.501	NA	.9
Other	3.677(0.0.853–163.643)		1.195 (0.074–19.384)	
Histological grade				
I	1.431 (0.224–9.134)	.705	2.366 (0.517–10.838)	.267
II	1.452 (0.855–2.467)	.167	1.501 (0.928–2.428)	.098
III	1.741 (1.226–2.473)	**.002**	1.609 (1.171–2.211)	**.003**
Subtype				
Luminal A	2.387 (0.268–21.218)	.435	0.622 (0.124–3.130)	.565
LuminalB (high Ki67)	2.386 (1.382–4.120)	**.002**	2.035 (1.209–3.427)	**.008**
Luminal HER2	1.138 (0.420–3.084)	.799	1.125 (0.448–2.823)	.802
HER2	1.763 (0.791–3.929)	.166	1.789 (0.893–3.584)	.101
TNBC	1.573 (1.022–2.424)	**.04**	1.551 (1.054–2.281)	**.026**
Chemotherapy				
Yes	1.673 (1.253–2.235)	**<.001**	1.644 (1.266–2.136)	**<.001**
No	0.897 (0.200–3.966)	.879	0.010 (0.000–1.902)	.085
Hormone therapy				
Yes	2.276 (1.529–3.390)	**<.001**	1.612 (1.013–2.566)	**.044**
No	16.272 (3.085–85.811)	**.001**	47.190 (2.813–791.662)	**.007**

BCS = breast-conserving surgery, CI = confidence interval, HER2 = human epidermal growth factor receptor 2, IDC = invasive ductal carcinoma, ILC = invasive lobular carcinoma, TNBC = triple-negative breast cancer.

## 4. Discussion

Pregnancy and childbirth are considered effective for preventing BC. However, studies have reported a poor prognosis for BC that develops 1 to 10 years after pregnancy.^[4–6]^ BC that occurs 1 to 2 years after pregnancy has the worst prognosis and is usually defined as PABC,^[7–9]^ although there are reports that the timing of pregnancy does not affect prognosis.^[5,10]^

PABC differs from non-PABC in terms of tumor biology and prognosis. PABC generally presents at an advanced stage. Regular BC screening in young women is rare, and breast congestion due to pregnancy and lactation may delay mass detection. Moreover, PABC is often associated with more aggressive subtypes than non-PABC, with higher rates of ER/PR-negative, HER2-positive, and TNBC subtypes.^[3,4,11–13]^ In recent years, surgery and chemotherapy are actively performed and the survival of patients with PABC has markedly improved. However, chemotherapy cannot be administered during the first trimester of pregnancy, and hormonal and radiation therapies are only possible after childbirth.

In the present study, the PABC group had more aggressive subtypes, such as TNBC and HER2, and a higher stage and histological grade than non-PABC group, consistent with results of previous studies. After PSM of variables that potentially affect prognosis, the survival of non-PABC patients decreased, as expected. However, patients with PABC showed a worse prognosis than matched non-PABC patients. These results confirm that pregnancy itself is a predictor of poor prognosis.

During pregnancy and lactation, the levels of hormones such as estrogen, progesterone, insulin-like growth factor 1, prolactin, and oxytocin increase, that can increase tumor cell proliferation.^[14,15]^ A weakened immune system during pregnancy and breast remodeling can also affect cancer growth and metastasis.^[12]^ However, despite several studies, the results are inconsistent owing to small population sizes and discrepancies in study methods.^[16]^ Therefore, the notion that PABC itself is associated with poor prognosis remains debatable.^[17]^

The prognosis of BC depends on the molecular subtype.^[13,18]^ Differences between BC subtypes may explain the inconsistency in results of several studies that analyzed the prognosis of patients with PABC. However, there have been few studies on the prognosis of PABC according to subtype. It has been reported that prognosis differs only for the luminal B subtype in PABC than in non-PABC group.^[12]^ The present study showed that patients with PABC with luminal B and TNBC subtypes had poor prognoses.

Efforts are being made to minimize BC treatment to achieve optimal outcomes, avoiding over- or under-treatment.^[19]^ A careful selection of treatment tailored for each patient can provide a better prognosis and quality of life and ensure low therapeutic toxicity. The St. Gallen International Expert Consensus 2017 debated on decreasing and increasing the extent of treatments for early stage BC.^[20]^ Consensus has been reached on several topics, such as tumor biology, subtypes, genomic signatures, and special populations, but PABC has not been included in the consensus due to insufficient data. The present study provides an important basis for personalized oncological treatment of patients with PABC. It is recommended to not reduce treatment for PABC. Moreover, an increased treatment should be considered for luminal B or TNBC subtypes for patients with PABC aged <35 years.

This study has some limitations. First, we failed to evaluate the role of the delay in initiating chemotherapy; however, Loibl et al have reported that survival rate is not affected by delayed chemotherapy in PABC.^[21]^ Second, this was a retrospective study as conducting randomized trial recruitment of pregnant women is not feasible. In contrast, the strength of this study is that it included the largest number of patients with PABC than those in previous studies. Additionally, this is the only study that matched age, stage, histological grade, subtype, and treatment, and the variables were corrected using PSM.

## 5. Conclusions

Patients with PABC have worse prognosis than non-PABC patients despite adjusting for age, stage, molecular subtype, histological type/grade, and chemotherapy. This effect was the most significant in patients with the luminal B and TNBC subtypes. An increase in treatment should be considered for patients aged <35 years with PABC, especially luminal B or TNBC subtypes. Further research on PABC biology and prognosis, and optimized treatment by subtype are warranted.

## Author contributions

**Conceptualization:** Hongki Gwak, Seong Hwan Kim.

**Data curation:** Hongki Gwak, Eun-Sook Lee, Min Ho Park, Seokwon Lee, Hyun Jo Youn, Seho Park, In Suck Suh.

**Formal analysis:** Hongki Gwak, Sang Seok Woo, Seong Hwan Kim.

**Funding acquisition:** Seong Hwan Kim.

**Investigation:** Hongki Gwak.

**Methodology:** Hongki Gwak, Sang Seok Woo, Seong Hwan Kim.

**Project administration:** Hongki Gwak.

**Resources:** Hongki Gwak.

**Software:** Hongki Gwak.

**Supervision:** Seong Hwan Kim.

**Validation:** Hongki Gwak.

**Visualization:** Hongki Gwak.

**Writing – original draft:** Hongki Gwak.

**Writing – review & editing:** Seong Hwan Kim.
